# Aspiration technique-based device is more reliable in cervical stiffness assessment than digital palpation

**DOI:** 10.1186/s12884-020-03080-x

**Published:** 2020-07-06

**Authors:** Sabrina Badir, Laura Bernardi, Francisco Feijó Delgado, Katharina Quack Loetscher, Gundula Hebisch, Irene Hoesli

**Affiliations:** 1 Pregnolia AG, Schlieren, Wiesenstrasse 33, 8952 Schlieren, Switzerland; 2 Clinic of Obstetrics, University Hospital Zurich, Zurich, Switzerland; 3 Gynecology and Obstetrics, Thurgau Hospital, Frauenfeld, Switzerland; 4 Department of Obstetrics and Gynecology, University Hospital Basel, Basel, Switzerland

**Keywords:** Uterine cervical consistency, Uterine cervical stiffness, Cervical ripening, Digital palpation, Preterm birth

## Abstract

**Background:**

The purpose of this study was to compare the reliability and reproducibility of the traditional qualitative method of assessing uterine cervical stiffness with those of a quantitative method using a novel device based on the aspiration technique.

**Methods:**

Five silicone models of the uterine cervix were created and used to simulate different cervical stiffnesses throughout gestation. The stiffness of the five cervix models was assessed both by digital palpation (firm, medium and soft) and with the Pregnolia System. Five self-trained participants conducted the device-based assessment, whereas 63 obstetricians and midwives, trained in digital palpation, conducted the cervical palpation.

**Results:**

The results of the two methods were analyzed in terms of inter-and intra-observer variability. For digital palpation, there was no common agreement on the assessment of the stiffness, except for the softest cervix. When assessing the same cervix model for a second time, 76% of the obstetricians and midwives disagreed with their previous assessment. In contrast, the maximum standard deviation for the device-based stiffness assessment for intra- and inter-observer variability was 3% and 3.4%, respectively.

**Conclusions:**

This study has shown that a device based on the aspiration technique provides obstetricians and midwives with a method for objectively and repeatably assess uterine cervical stiffness, which can eliminate the need to rely solely on a subjective interpretation, as is the case with digital palpation.

## Background

Appropriate mechanical functioning of the uterine cervix, the cervical competence, is critical for maintaining pregnancy until term and allowing the fetus to mature [1, 2]. For delivery at term the cervix must soften, shorten and fully dilate during the latent first and second stage of labor [3, 4].

Cervical length, cervical consistency or softness, and cervical dilatation are three clinical parameters used to describe cervical ripening throughout pregnancy and to predict time of delivery [1, 5, 6]. Softening is related to changes in collagen content and organization, structural cervical changes, an increase of water content, and concentration of proteoglycans in the extracellular matrix [1, 7, 8]. Cervical softening can already be detected in the first month after fertilization [9], and continues progressively throughout pregnancy [9–15], while cervical length remains stable until it gradually shortens during the third trimester [16]. Cervical dilatation generally starts with labor [11], with delivery being preceded by complete cervical softening, shortening and dilatation [5].

Predicting timing of delivery plays an important role in prenatal care. Anticipating whether a birth may occur preterm allows for clinical interventions that can delay prematurity [17–20], or accelerate fetal development [21], and currently this assessment relies heavily on determining the length of the cervix [20]. Timing the delivery can also be relevant in the success of induction of labor. Presently, clinicians assess cervical maturation using the Bishop Score to determine the need for cervical ripening prior to induction. A softer, shorter and more dilated cervix is associated with a shorter time to labor onset, as well as a smaller risk for a failed induction and a cesarean section due to cervical dystocia [10, 22] Cervical dystocia happens when the cervical ripening does not occur at term and the cervix does not shorten and dilate. If, however, cervical ripening occurs too fast (cervical incompetence) there is a higher risk for preterm birth [23]. Ultimately, more accurately predicting delivery timing can reduce levels of neonatal morbidity and mortality. The ability of the cervix to fulfil its different roles throughout pregnancy is fundamental to ensure a timely and successful delivery, and therefore there is strong clinical interest in evaluating its condition [10].

By digitally palpating the cervix during a pelvic exam, cervical status can be evaluated by its tissue stiffness, its length and its dilatation [5, 22, 24]. With the introduction of ultrasound, cervical length and cervical dilation to an extent became objectively quantifiable parameters for estimating the risk of preterm delivery [25]. Cervical softness, however, remained a subjective evaluation by the obstetrician or the midwife, dependent on the experience of the examiner [5]. There is no well-established objective technique to assess the cervical softness during pregnancy. Different ultrasound-based elastography methods have lately been applied in clinical trials to study cervical stiffness in pregnancy. However, these methods have shortcomings, namely the characterization of the applied force, leading to operator dependency, or the limitations on the assumptions made about the biomechanical properties of the cervical tissue, which do not allow a clear interpretation of the results, leading to a lack of a clear cut-off value for predicting preterm birth [26].

In this study, we compare inter-observer and intra-observer variability of two methods to assess cervical stiffness: i) digital palpation, and ii) a new device based on the aspiration technique (Pregnolia System).

## Methods

For this study, five silicone models of the uterine cervix were used to simulate different cervical stiffnesses. These models were used as test samples for the two cervical stiffness assessment methods described in this section.

### Production of the cervix models

Five silicone cervix models were produced using a two-component platinum silicone rubber gel (EcoFlex™ GEL, Smooth-On Inc.), with Shore hardness of 000–35. The two components were mixed by hand in a 1:1 ratio by weight. A small amount of Flesh (PMS 488C) and Red (PMS 186C) pigments (Silc Pig™, Smooth-On Inc.) was added to the material and mixed by hand, to color the model.

To achieve different stiffnesses, a softener (Slacker®, Smooth-On Inc.) was added to the mixture at different ratios: 0% (pure EcoFlex™ Gel), 10%, 20%, 35%, and 65%.

**Fig. 1:**
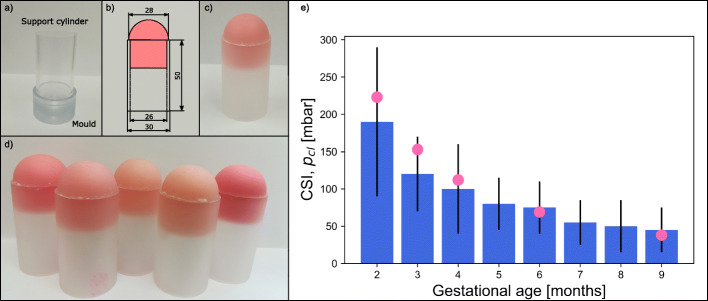
**a**) Materials for silicone production: support cylinder and mold; **b**) dimensions of the support cylinder and of the cervix model; **c**) cervix model; **d**) five cervix models produced **e**) Cervical Stiffness Index (p_cl_ in mbar) at different gestational ages (from Badir et al., 2013 [10], blue bars, mean ± standard deviation) and closing pressure of the five silicone cervices produced (pink dots)

These mixtures were then thoroughly mixed and poured into Plexiglas molds (Fig. 1a) previously treated with a releasing agent (Ease Release™ 200, Mann Release Technologies) according to the datasheet. This treatment was necessary to easily release the models from the mold.

The Plexiglas mold partially resembled the shape of the cervix, producing a half-sphere with a diameter of 28 mm and a small hole in the center to simulate the cervical canal (Fig. 1b and c). A Plexiglas cylinder (outer diameter 30 mm, inner diameter 26 mm and height 50 mm), inserted in the mold served as support for the silicone cervix (Fig. 1b and c).

The silicone was then cured for at least 2 h at room temperature. Once cured, the silicone cervices were gently removed from the mold and their surfaces were covered with talcum powder to avoid stickiness (Fig. 1d).

The five cervix models were produced to obtain a range of cervical stiffness values that resemble the ones of the human cervix during the second (weeks 5–8), third (weeks 9–12), fourth (weeks 13–17), sixth (weeks 22–25) and ninth (weeks 36–40) months of gestation, according to the values obtained in vivo by Badir et al. [10], see Fig. 1e.

### Pregnolia System

The Pregnolia System is a new device used to assess the stiffness of the uterine cervix. The procedure is based on the aspiration technique, as described in [10, 27, 28].

**Fig. 2:**
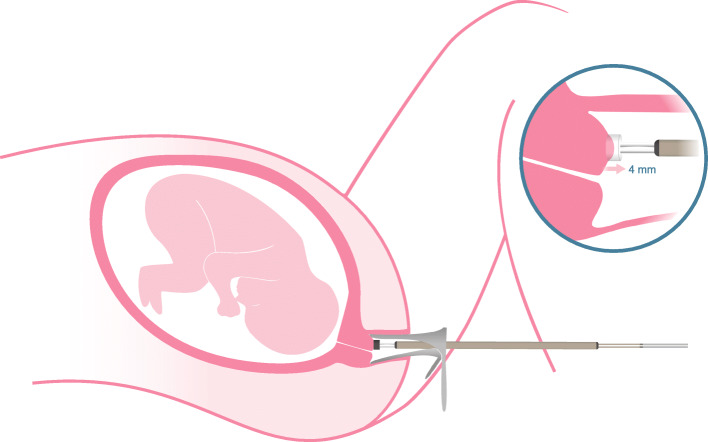
The Pregnolia System is used by placing the probe directly on the uterine cervix. The tissue is gently pulled into the probe tip by a fixed distance

Briefly, the device is composed of two elements: (i) a control unit containing a pump, which creates a vacuum following a defined negative pressure versus time curve; and (ii) a single-use sterile probe (Fig. 2) applied on the cervix through a speculum. As soon as a tight contact between the probe tip and the anterior lip of the cervix is established, the tissue is slowly and gently pulled into the tip until it touches the ceiling of the tip’s cylindrical cavity. The vacuum pressure needed to achieve this 4 mm displacement is the closing pressure (p_cl_), which is a proxy value for cervical stiffness and is called Cervical Stiffness Index (CSI).

A prototype of the Pregnolia System has been previously used in a clinical trial to assess the cervical stiffness of 50 pregnant and 50 non-pregnant women, as reported by Badir et al. [10].

### Pregnolia System test protocol and analysis

**Fig. 3:**
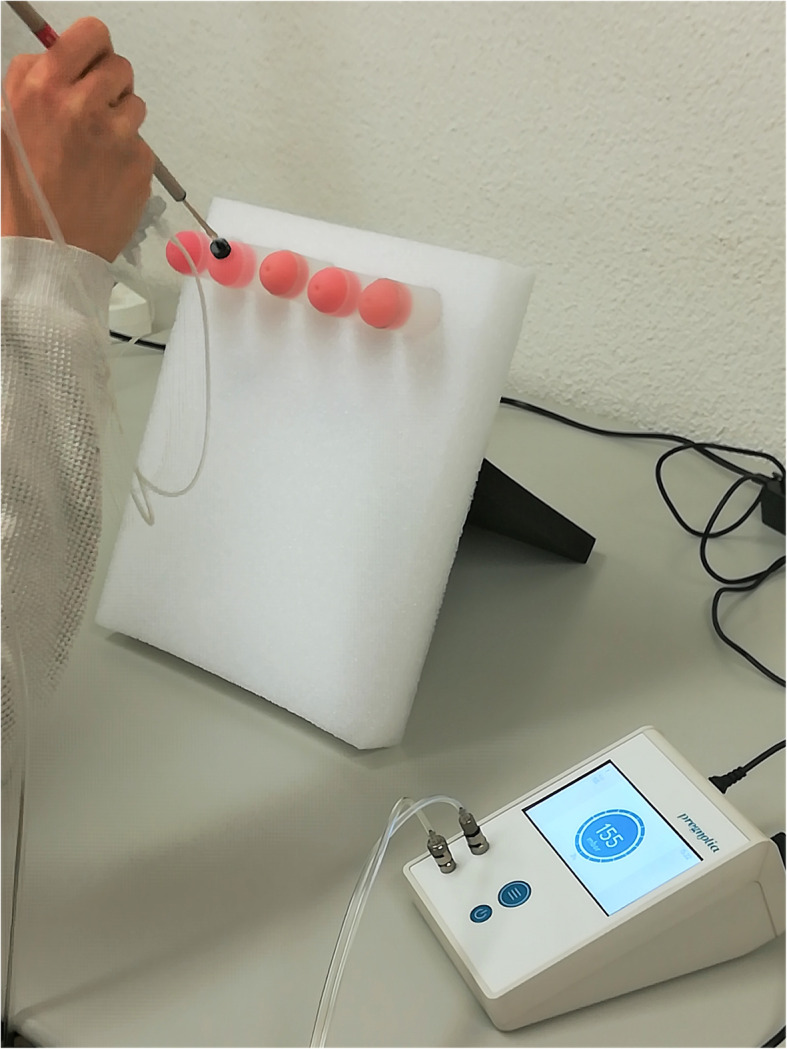
Pregnolia System test. The stiffness of the five cervices is assessed using the Pregnolia System by each participant at three different time points

Five self-trained participants measured the stiffness of the five cervix models using the Pregnolia System (Fig. 3). Each participant conducted stiffness measurements on all five cervices at 9 am, 12 pm and 3 pm (T1, T2, T3). This led to a total of three measurements per cervix per participant and a total of 15 measurements per cervix. All measurements were conducted on the same location with the same measurement procedure, using the same measuring device. Participants measured all five cervices over a short period of time.

Results were analyzed in terms of inter- and intra-observer variability [29] and are reported as mean (σ) and standard deviation (μ). The relative standard deviation (RSD) was calculated to expresses how tightly the data are clustered around the mean value, with a small relative standard deviation indicating high precision.

### Digital palpation test protocol and analysis

For this test, 63 participants were selected: 33 obstetricians and 30 midwives, all trained in performing cervical palpation. Among those, 61% of the obstetricians and 73% of the midwives stated they perform cervical palpation routinely. Each participant was asked to categorize the stiffness of the silicone cervices as firm, medium or soft. They were sequentially given eight cervices to assess, first receiving each of the five cervices in a random order, and subsequently, without their knowledge, three repetitions, selected at random.

Results were analyzed in terms of inter- and intra-observer variability [29]. For assessing the reliability of the rating among participants, we computed Fleiss’ kappa [30] for the first rating of each of the five silicone cervices, i.e. excluding repetitions. Where reported, statistical significance was calculated with a Mann-Whitney U test, *p*-value < 0.05.

### Ethics

Ethics approval for this study is deemed not necessary according to national legislations (Human Research Act 810.30, see “Declarations” for more details).

## Results

### Pregnolia System test

**Fig. 4:**
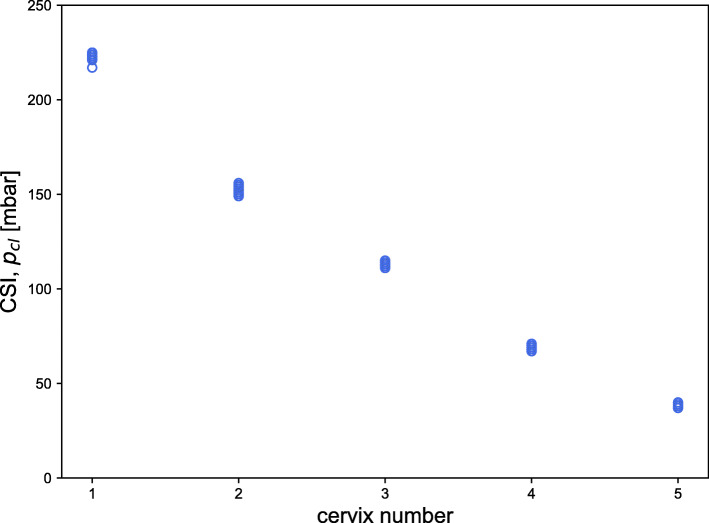
Results of the assessment made using the Pregnolia System, showed as Cervical Stiffness Index (p_cl_ in mbar). *n* = 15 for each cervix

Figure 4 shows the results obtained as closing pressure (p_cl_) in mbar. Cervix models are reported from the stiffest (*cervix 1*) to the softest one (*cervix 5*). For each cervix, 15 data points are reported.

#### Intra-observer variability

Table 1 reports the results obtained for the intra-observer variability test for each of the five participants. Each participant assessed the stiffness of the five cervix models 3 times (9 am, 12 pm, 3 pm). Results are reported as mean (μ) ± standard deviation (σ) and as relative standard deviation (RSD). The maximum relative standard deviation was 3%.

**Table 1:** Results of the intra-observer variability test

	Participant 1		Participant 2		Participant 3		Participant 4		Participant 5	
Cervix No.	μ ± σ [mbar]	RSD [%]	μ ± σ [mbar]	RSD [%]	μ ± σ [mbar]	RSD [%]	μ ± σ [mbar]	RSD [%]	μ ± σ [mbar]	RSD [%]
1	223.3 ± 1.5	0.7	223.0 ± 1.0	0.4	223.0 ± 1.7	0.8	224.0 ± 1.0	0.4	221.0 ± 3.6	1.6
2	154.3 ± 0.6	0.4	152.7 ± 1.5	1.0	153.0 ± 1.0	0.7	155.7 ± 0.6	0.4	151.0 ± 2.6	1.8
3	112.7 ± 0.6	0.5	113.0 ± 0.0	0.0	111.7 ± 0.6	0.5	113.7 ± 0.6	0.5	112.7 ± 2.1	1.8
4	68.7 ± 1.5	2.2	68.7 ± 0.6	0.8	68.3 ± 0.6	0.8	70.0 ± 1.0	1.4	69.0 ± 1.0	1.5
5	38.7 ± 1.2	3.0	37.7 ± 0.6	1.5	38.0 ± 1.0	2.6	38.7 ± 0.6	1.5	37.0 ± 0.0	0.0

#### Inter-observer variability

Table 2 reports the results obtained for the inter-observer variability test, stated per time point. The stiffness of each model was assessed five times during each time point, once per participant. Results are reported as mean (μ) ± standard deviation (σ) and as relative standard deviation (RSD). The maximum relative standard deviation observed was 3.4%.

**Table 2:** Results for the inter-observer variability test

	T1		T2		T3	
Cervix No.	μ ± σ [mbar]	RSD [%]	μ ± σ [mbar]	RSD [%]	μ ± σ [mbar]	RSD [%]
1	222.2 ± 2.9	1.3	223.2 ± 1.3	0.6	223.2 ± 1.6	0.7
2	153.4 ± 2.5	1.6	152.8 ± 2.4	1.6	153.8 ± 1.5	1.0
3	112.6 ± 1.1	1.0	112.6 ± 0.9	0.8	113.0 ± 1.4	1.3
4	69.2 ± 0.8	1.2	69.2 ± 1.3	1.9	68.4 ± 0.9	1.3
5	38.2 ± 0.8	2.1	38.2 ± 1.3	3.4	37.6 ± 0.5	1.5

### Digital palpation test

#### Inter-observer variability

**Fig. 5:**
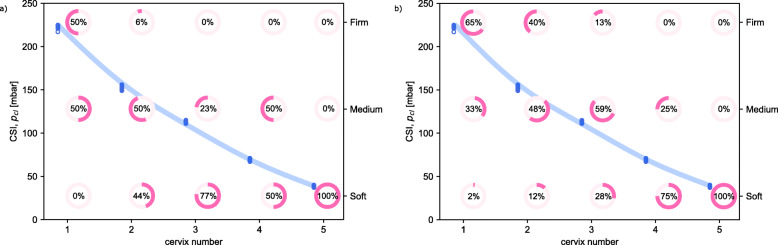
**a**) Inter-observer variability results of the assessment of the first cervix for each participant (*n* = 6, 16, 13, 18, 10, respectively) Blue dots represent the assessment made using the device (as in Fig. 4). Pink circles indicate the percentage of participants giving the corresponding assessment (firm/medium/soft); **b**) Inter-observer variability results of the assessment of all the cervices (*n* = 111, 105, 101, 104, 83, respectively). Blue dots represent the assessment made using the device (as in Fig. 4). Pink circles indicate the percentage of participants giving the corresponding assessment (firm/medium/soft)

Figure 5a shows the results of the assessment of the first silicone model presented to each participant. When assessing a cervix model for the first time, the participants did not have any reference and therefore their judgements were not influenced by other parameters, such as the comparison with previous models.

As shown in the figure, only the softest cervix (number 5) was given the same stiffness assessment by all participants, with no common rating for all the other cervices: 50% of the participants assessed *cervix 1* as firm, and the remaining 50% as medium; participants assessed *cervix 2* as firm (6%), medium (50%) and soft (44%); the stiffness of *cervix 3* was considered by the participants medium (23%) or soft (77%) and *cervix 4* was either medium (50%) or soft (50%). Note that the number of participants who assessed each cervix first varies: 6 participants assessed *cervix 1* as first, 16 *cervix 2*, 13 *cervix 3*, 18 *cervix 4* and 10 *cervix 5*.

To quantify the agreement reliability between the different raters, we computed a Fleiss’ kappa coefficient of 0.321 (95% confidence interval 0.317–0.325, *p*-value < 0.05), indicating only a fair level of agreement, according to the Altman classification (poor, fair, moderate, good and very good) [31].

Figure 5b reports all the results obtained. As for the previous results, *cervix 5* was judged soft by all the participants. The assessment for *cervices 1, 2 and 3* was split among all the three possibilities, whereas 75% of participant judged *cervix 4* as soft, and the remaining 25% as medium.

Results were also assessed by splitting the participants into two categories: obstetricians and midwives (Table 3). No statistically significant differences were observed in the assessment of the stiffness when comparing the two categories.

**Table 3:** Assessment split for the two categories: obstetricians (O) and midwives (M)

Cervix No.	Firm		Medium		Soft		*p*-value
	O	M	O	M	O	M	
1	63%	67%	33%	33%	4%	0%	0.3
2	44%	37%	43%	51%	13%	12%	0.2
3	9%	17%	60%	60%	31%	23%	0.1
4	0%	0%	21%	29%	79%	71%	0.2
5	0%	0%	0%	0%	100%	100%	n/a

#### Intra-observer variability

When assessing the same cervix model for a second time, only 24% of the participants did not change their previous assessment on any of the three repeated models. 44% of the participants changed the assessment of one model, 27% of two models and 5% of the participants changed the assessment of all three repeated models. In four cases, the assessment was changed from soft directly to stiff (once for *cervix 1*, twice for *cervix 2*, and once for *cervix 3*).

Table 4 reports the changes in the assessment of the stiffness. There were 189 total repetitions (63 participants, 3 repetitions each) and participant assessment changed 37% of the time. Among the changes, 21% were from a higher to a lower stiffness assessment, 79% from a lower to a higher. When split for categories (obstetricians and midwives), there were 42% changes among the repetitions of the obstetricians and 31% among the repetitions of the midwives (differences not statistically significant). The repeated cervices were evenly distributed among categories.

**Table 4:** Changes in the assessment of the stiffness

	Change from higher to lower stiffness	Change from lower to higher stiffness	Total changes (out of total repetitions)
**All**	21%	79%	37%
**Obstetricians**	17%	83%	42%
**Midwives**	29%	71%	31%

#### Distribution of the stiffness assessment

**Fig. 6:**
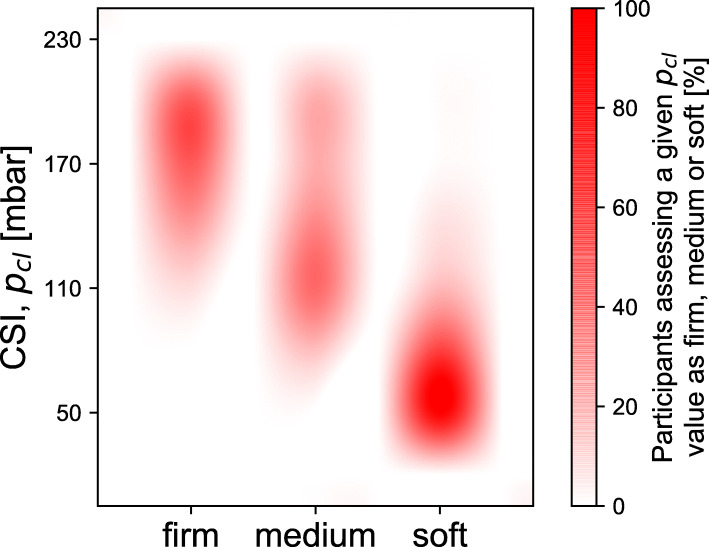
Each colored patch shows the interpolated range of the stiffness values (in mbar) of the silicone cervices, assessed by the participants through palpation as firm, medium or soft. The distributions indicate the broad and overlapping range of stiffnesses encompassed by each classification. For instance, most participants assess cervices with low p_cl_ value stiffnesses as soft, however, the assessment of soft includes cervices with p_cl_ values around 120 mbar, which were also assessed both as medium and soft

Figure 6 shows the distribution of the stiffness assessment in terms of closing pressure (p_cl_) based on digital palpation. As shown in the plot, a cervix assessed as “firm” by digital palpation can have a stiffness varying from ~ 110 to ~ 230 mbar. A cervix classified as medium can vary from ~ 70 to ~ 230 mbar. A soft cervix can have a stiffness in the range of ~ 35 to ~ 150 mbar.

## Discussion

In this study, the inter- and intra-observer variability of digital palpation and of the aspiration technique as methods for assessing cervical stiffness were analyzed and compared.

The results clearly demonstrate that digital palpation is an unreliable method to assess cervical stiffness. The method is subjective, but, to our knowledge, reliability has never been quantified. Results reported in Fig. 5 clearly show that digital palpation is not a sufficiently reproducible method, since different participants assessed the stiffness of the same cervices differently. Furthermore, this method is also not reliably repeatable, since when the same participants were asked to assess the stiffness of the same cervix, only 24% did not change their previous assessment at all. 44% of the participants changed the assessment at least once and 5% changed the assessment of all three the repeated cervices. Furthermore, when analyzing the aggregate data, it is patently observable that each of the traditional descriptors encompasses a wide range of actual stiffnesses, with intermediate stiffness levels being in fact described as soft, medium and stiff.

On the contrary, the aspiration technique-based device is a repeatable and reproducible method to assess the cervical stiffness, as demonstrated by the extremely low relative standard deviation calculated and the results reported in Fig. 4. The results also show the possibility of distinguishing much smaller differences in tissue stiffness compared to digital palpation, which poorly differentiates close stiffness values. This new technique could help obstetricians and midwives assess cervical stiffness in an objective and repeatable way without the need to rely on their own judgment.

Noteworthy, the aspiration technique-based device requires the use of a speculum during the examination, contrary to digital palpation. Speculum application is a common practice in the field of gynecology and obstetrics. Speculum-based examinations may be unpleasant for the women, however, also digital palpation may lead to discomfort and embarrassment for the woman [32].

Interestingly, a few participants commented that the stiffest cervix model, with the equivalent stiffness corresponding to gestational weeks 5–8, was, in their opinion, not representative of a stiff cervix. This can also be seen in Table 4: the majority of the changes were from a lower to a stiffer value, as both obstetricians and midwives initially judged the models softer than what they did at the end, after assessing several models. As reported by Badir et al. [10], a cervix of a non-pregnant woman can be more than twice as stiff as *cervix* 1, but we deliberately chose not to create a stiffer cervix since the Bishop score method was initially developed to assess the stiffness of women close to labor, when the cervix is very soft (see Fig. 1e, weeks 36–40 of gestation). Given the fact that the division among stiff, medium and soft is made close to labor, we anticipated that a stiffness of ~ 220 mbar, corresponding to a cervix at gestational weeks 5–8, would be far stiffer than what is normally assessed by digital palpation in women close to labor.

The strength of the study lies in the innovative, reproducible non-invasive method for analyzing cervical consistency and the large number of participants assessing cervical stiffness. However, the primary limitation of this study is due to the fact that stiffness was measured on silicone models and not in vivo on actual cervices. Cervical tissue in pregnancy is not homogenous in the anterior and posterior part and depends on maternal factors (parity, weight, age). While there is no reason to believe that human operator objectiveness would increase in vivo*,* these conclusions would gain by the performance of a similar study in vivo, where the performance of the device in real tissue can be measured. Due to this fact, we cannot directly compare this method to Bishop score, or assess outcome prediction. Furthermore, the device does not analyze the full depth of the cervical tissue, however previous comparison to a method that measures tissue stiffness on the whole cervix showed equivalent results [33]. Some of the participants reported that the models feel different from real cervices, noting that there is no mucus and the shape of the model cervix is only partially representative of the real one, preventing them from palpating the lateral side of the cervix. Nevertheless, as shown in Fig. 1e, the stiffness of the silicone models is representative of the physiological cervices [10].

## Conclusion

This study has shown that an aspiration technique-based device provides obstetricians and midwives with a method for objectively and repeatably assessing uterine cervical stiffness, eliminating sole reliance on subjective interpretations from digital palpation.

## Data Availability

The datasets used and/or analysed during the current study are available from the corresponding author on reasonable request.

## Abbreviations

CSI: Cervical stiffness index
P_cl_: Closing pressure
